# Efficacy of commercial products on nursery pig growth performance fed diets with fumonisin contaminated corn

**DOI:** 10.1093/tas/txaa217

**Published:** 2020-11-21

**Authors:** Zhong-Xing Rao, Mike D Tokach, Steve S Dritz, Jason C Woodworth, Joel M DeRouchey, Robert D Goodband, Hilda I Calderon

**Affiliations:** 1 Department of Animal Sciences and Industry, College of Agriculture, Kansas State University, Manhattan, KS; 2 Department of Diagnostic Medicine/Pathobiology, College of Veterinary Medicine, Kansas State University, Manhattan, KS; 3 Department of Statistics, College of Arts and Sciences, Kansas State University, Manhattan, KS

**Keywords:** fumonisin, growth, mycotoxin, nursery pigs, sphinganine, sphingosine

## Abstract

Two experiments were conducted to determine the efficacy of various commercial products on growth performance of nursery pigs fed diets high in fumonisin. In experiment 1, 350 pigs (241 × 600; DNA, Columbus, NE; initially 9.9 kg) were used with five pigs per pen and 14 replicates per treatment. After weaning, pigs were fed common diets for 21 d before the experiment started. The five dietary treatments consisted of a positive control (low fumonisin), a negative control (60 mg/kg of fumonisin B1 + B2 in complete diet), and the negative control with one of three products (0.3% of Kallsil Dry, Kemin Industries Inc., Des Moines, IA; 0.3% of Feed Aid Wide Spectrum, NutriQuest, Mason City, IA; 0.17% of Biofix Select Pro, Biomin America Inc., Overland Park, KS). Diets were fed in mash form for 14 d and followed with a low fumonisin diet for 13 d. For the 14-d treatment period, pigs fed the positive control diet and Biofix Select Pro had greater (*P* < 0.05) average daily gain (ADG), average daily feed intake (ADFI), and gain:feed (G:F) compared to those fed the high fumonisin negative control, or high fumonisin diets with Kallsil Dry or Feed Aid Wide Spectrum. Serum sphinganine to sphingosine ratios (SA:SO) were greater (*P* < 0.05) in all pigs fed high fumonisin diets compared to the positive control. In experiment 2, 300 pigs (241 × 600; DNA; initially 10.4 kg) were used. Procedures were similar to experiment 1 except there were 12 replicate pens per treatment, high fumonisin diets contained 30 mg/kg fumonisin, and experimental diets were fed for 28 d. Similar to experiment 1, pigs fed the positive control diet and treatment with Biofix Select Pro had greater (*P* < 0.05) ADG and G:F, and lower (*P <* 0.05) serum SA:SO compared to pigs fed the high fumonisin negative control, or high fumonisin diets with Kallsil Dry or Feed Aid Wide Spectrum. In summary, pigs fed diets containing 60 mg/kg of fumonisin for 14 d or 30 mg/kg of fumonisin for 28 d had poorer ADG and G:F and greater serum SA:SO compared to pigs fed a diet with less than 5 mg/kg of fumonisin. Adding Biofix Select Pro to diets appeared to mitigate the negative effects of high fumonisin concentrations, while Kallsil Dry and Feed Aid Wide Spectrum did not.

## INTRODUCTION

Fumonisins are a group of toxic secondary metabolites produced mostly by *Fusarium vertcilliodes* and *F. proliferatum*. Fumonisins are highly polar and soluble in water. The fumonisins that cause concern in the animal feed industry are fumonisin B1, B2, and B3, and their concentrations generally appear in an approximate ratio of 3:1:1. In contaminated feed ingredients, fumonisin B1 is most toxic to animals, while fumonisin B2 is secondary, with B3 the least toxic. Fumonisin contamination has been increasing in grains used for animal feed in the United States (Hendel et al., 2020). Fumonisin disrupts synthesis of complex sphingolipids, which results in cell damage and the accumulation of sphinganine (SA) and sphingosine (SO), the precursors of sphingolipids, in tissue. Sphinganine accumulates at a faster rate than SO. Therefore, serum SA:SO ratio has been used as a biomarker to determine the severity of fumonisin intoxication (Zomborszky et al., 2000; Hsiao et al., 2007; Dilkin et al., 2010). Fumonisin toxicosis reduces growth performance and causes damage to intestinal structure, liver, kidneys, and lung (Bracarense et al., 2012; Ensely and Radke, 2019). In a previous study, pigs fed increasing amounts of naturally contaminated fumonisin corn (fumonisin B1 + B2 levels of 7.2 to 35.1 mg/kg) resulted in a linear reduction in growth performance and increased serum SA:SO ratio (Rao et al., 2020). In addition, a SA:SO ratio of over 1:1, which is generally considered as fumonisin intoxication, was observed in pigs fed 32.7 mg/kg fumonisin or greater.

Several commercial products have the potential to mitigate the effects of fumonisin, but little in vivo research is available to verify their efficacy. Even though there is no commercial product that is FDA approved for its ability to bind fumonisin, clay-based products are sometimes added to diets to hopefully mitigate the negative effect of fumonisin. Hence, we conducted these experiments to determine the efficacy of commercial products on nursery pig growth performance and serum SA:SO ratio when fed diets with high fumonisin contamination.

## MATERIALS AND METHODS

The Kansas State University Institutional Animal Care and Use Committee approved the protocols (protocol number: 4036.25) used in these experiments and they were conducted at the Kansas State University Swine Teaching and Research Center in Manhattan, KS. Each pen (1.2 m × 1.2 m) provided 0.28 m^2^ per pig and was equipped with a 4-hole, dry self-feeder and a nipple waterer to provide ad libitum access to feed and water.

In both experiments, pigs were weaned at approximately 21 d of age and placed in pens of five pigs based on initial BW and gender. A common phase 1 pelleted diet was fed for 7 d and a common phase 2 mash diet was fed for another 14 d. At d 21 after weaning, which was considered d 0 of the trial, pens of pigs were randomly allotted to treatment in a randomized complete block design with BW as the blocking factor. Diet samples were collected and analyzed for mycotoxins (Tables 1 and 2). Diets from experiment 1 were analyzed for all major mycotoxins without and with a 100× sample dilution, and diets from experiment 2 were analyzed twice with 100× sample dilution at the North Dakota State University Veterinary Diagnostic Laboratory (NDSU, Fargo, ND). Diets from both experiments were also analyzed with 10× sample dilution for fumonisin at Trilogy laboratory (Washington, MO). Both NDSU and Trilogy laboratories utilized a 10 mg/kg fumonisin standard curve. Diluting samples was required for the high fumonisin levels to be within the standard curve used in each of the laboratories.

**Table 1.** Dietary mycotoxin levels, experiment 1 (as-fed basis, mg/kg)^*a*^

			Negative control with		
Item	Positive control	Negative control	Kallsil Dry^*b*^	Feed Aid Wide Spectrum^*c*^	Biofix Select Pro^*d*^
Fumonisin B1					
NDSU (undiluted)	2.33	49.36	48.43	49.39	50.48
NDSU (diluted 100×)	3.07	46.76	42.95	47.89	40.58
Trilogy (diluted 10×)	1.80	35.10	36.80	37.00	37.00
Fumonisin B2					
NDSU (undiluted)	0.57	11.95	12.13	12.15	12.70
NDSU (diluted 100×)	0.97	11.44	11.17	12.92	11.22
Trilogy (diluted 10×)	0.10	5.90	6.70	7.10	6.90
Fumonisin B3					
Trilogy (diluted 10×)	0.20	4.20	4.50	4.80	4.60

^*a*^A representative sample of each diet was collected from every fifth bag of feed manufactured for each treatment. All mycotoxins were analyzed at NDSU (Fargo, ND) by LC/MS/MS. The standard curve for fumonisin was up to 10 ppm. Fumonisin B1, B2 and B3 were analyzed at Trilogy lab (Washington, MO) by LC/MS/MS. The standard curve for fumonisin was up to 10 ppm. Aflatoxin B1, aflatoxin B2, aflatoxin G1, aflatoxin G2, T-2 Toxin, ochratoxin, and sterigmatocystin were below detectable concentration (<20 ug/kg); HT-2 toxin and vomitoxin were below detectable concentration (<200 ug/kg); and zearalenone was below detectable concentration (<100 ug/kg).

^*b*^Kallsil Dry (Kemin Industries Inc., Des Moines, IA).

^*c*^Feed Aid Wide Spectrum (NutriQuest, Mason City, IA).

^*d*^ Biofix Select Pro (Biomin America Inc., Overland Park, KS).

**Table 2.** Dietary mycotoxin levels, experiment 2 (as-fed basis, mg/kg)^*a*^

			Negative control with		
Item	Positive control	Negative control	Kallsil Dry^*b*^	Feed Aid Wide Spectrum^*c*^	Biofix Select Pro^*d*^
Fumonisin B1					
NDSU 1st	4.10	24.96	25.10	21.41	20.59
NDSU 2nd	3.24	23.83	23.73	21.26	23.45
Trilogy	2.70	18.90	19.10	17.30	19.20
Fumonisin B2					
NDSU 1st	1.07	6.06	7.31	5.17	6.52
NDSU 2nd	0.96	6.65	7.06	5.63	6.53
Trilogy	0.50	4.40	4.90	3.60	4.70
Fumonisin B3					
Trilogy	0.40	2.70	2.60	2.10	2.60

^*a*^A representative sample of each diet was collected from 24 bags of feed for each treatment. All mycotoxins were analyzed at NDSU (Fargo, ND) by LC/MS/MS. The standard curve for fumonisin was up to 10 ppm. Fumonisin B1, B2, and B3 were analyzed at Trilogy lab (Washington, MO) by LC/MS/MS. The standard curve for fumonisin was up to 10 ppm. Aflatoxin B1, aflatoxin B2, aflatoxin G1, aflatoxin G2, T-2 Toxin, ochratoxin, and sterigmatocystin were below detectable concentration (<20 ug/kg); HT-2 toxin and vomitoxin were below detectable concentration (<200 ug/kg); and zearalenone was below detectable concentration (<100 ug/kg).

^*b*^Kallsil Dry (Kemin Industries Inc., Des Moines, IA).

^*c*^ Feed Aid Wide Spectrum (NutriQuest, Mason City, IA).

^*d*^Biofix Select Pro (Biomin America Inc., Overland Park, KS).

The multiple mycotoxin assay conducted at North Dakota State University Veterinary Diagnostic Laboratory was based on an Agilent Technologies (Santa Clara, CA) method for mycotoxin in corn using ultrahigh pressure liquid chromatography and tandem mass spectrometric detection (UHPLC/MS/MS) with modifications (Varga et al., 2013). The detail of this analysis can be found in Rao et al. (2020). The fumonisin analysis conducted at Trilogy laboratories was an internally validated method for 22 mycotoxins, including fumonisin, using LC/MS/MS. For fumonisin analysis, the samples were ground using Retsch GM200 knife mill with serrated blades. The samples were then extracted using official AOAC extractions method (method 995.15; AOAC Int., 2019) with methanol/water (25 g/100 mL, v/v) solution and shook on a laboratory orbital shaker for 1 h. The samples were then filtered with a Whatman No. 1 filter. Extracts were diluted with 1% acetic acid in a 1:4 ratio. Matrix calibration curve was prepared using a known nondetected sample following the above procedure and fortification. The samples were then injected and analyzed on Sciex 6500 Qtrap (AB Sciex LLC, Framingham, MA).

In experiment 1, a total of 350 pigs (241 × 600; DNA, Columbus, NE; initially 9.9 kg) were used in a 27-d growth trial. There were five pigs per pen and 14 replicates per treatment. The five dietary treatments consisted of a positive control (low fumonisin diet, 3 to 4 mg/kg fumonisin B1 + B2), a negative control (approximately 50 to 60 mg/kg of fumonisin B1 + B2), and three other treatments as negative control with one of three different commercial products (Kallsil Dry, Kemin Industries Inc., Des Moines, IA; Feed Aid Wide Spectrum, NutriQuest, Mason City, IA; Biofix Select Pro, Biomin America Inc., Overland Park, KS; Table 3). The product inclusion levels were provided by their respective suppliers and were 0.3% for Kallsil Dry and Feed Aid Wide Spectrum and 0.17% for Biofix Select Pro. High fumonisin diets were fed in mash form for 14 d and followed with a low fumonisin (5 mg/kg) common mash diet for 13 d as a posttreatment period. Two diets were formulated using control corn (approximately 5 mg/kg of fumonisin B1 + B2) or fumonisin-contaminated corn (approximately 80 mg/kg of fumonisin B1 + B2). The diet manufactured with low fumonisin corn was used as the positive control. The diet manufactured with high fumonisin-contaminated corn was manufactured as a single large batch of basal diet. During the bag-off process, each bag was stacked sequentially on 12 numbered pallets to allow fumonisin to be evenly distributed in all treatment diets. Three pallets of bags were randomly selected for each of the four fumonisin treatments. Each treatment’s basal diet was then mixed again with sand or one of the three commercial products to produce the final treatment diet. These experimental diets were fed during the 14-d treatment period. Meanwhile, a low fumonisin diet for the 13-d post treatment period was formulated and manufactured identically to the positive control diet that was used in the 14-d treatment period. Representative diet samples were obtained from every fifth bag of feed manufactured. Pen weights and feed disappearance were measured on d 0, 7, 14, 21, and 27 to determine ADG, ADFI, and G:F.

**Table 3.** Diet composition (as-fed basis)^*a*^

Item	Positive control	Negative control^*b*^
Ingredients, %		
Corn, 5 mg/kg of fumonisin	64.70	–
Corn, 80 mg/kg of fumonisin	–	64.48
Soybean meal	28.00	28.00
Soybean oil	3.00	3.00
Monocalcium phosphate	0.85	0.85
Calcium carbonate	0.75	0.75
Sodium chloride	0.60	0.60
l-Lysine HCl	0.55	0.55
dl-Methionine	0.21	0.21
l-Threonine	0.23	0.23
l-Tryptophan	0.06	0.06
l-Valine	0.16	0.16
Vitamin premix^*c*^	0.25	0.25
Trace mineral premix^*c*^	0.15	0.15
Phytase^*d*^	0.08	0.08
Sand	–	0.30
Total	100	100
Calculated analysis		
Standardized ileal digestible amino acids, %		
Lysine	1.30	1.30
Isoleucine:lysine	53	53
Leucine:lysine	111	111
Methionine:lysine	36	36
Methionine and cysteine:lysine	56	56
Threonine:lysine	63	63
Tryptophan:lysine	20.0	20.0
Valine:lysine	69	69
Histidine:lysine	35	35
Net energy, kcal/kg	2,534	2,527
Crude protein, %	19.8	19.8
Calcium, %	0.61	0.61
STTD P^*e*^, %	0.44	0.44

^*a*^Diets were fed during the 14-d treatment period. During the 13-d posttreatment period, the positive control was fed to all groups of pigs.

^*b*^The three high fumonisin treatments containing commercial products were manufactured by using the negative control with product added at the expense of sand. Inclusion rates were 0.3% of Kallsil Dry (Kemin Industries Inc., Des Moines, IA), 0.3% of Feed Aid Wide Spectrum (NutriQuest, Mason City, IA), and 0.17% of Biofix Select Pro (Biomin America Inc., Overland Park, KS).

^*c*^Provided per kg complete feed: 4,133 IU vitamin A; 1653 IU vitamin D3; 44 IU vitamin E; 3 mg vitamin K; 0.03 mg vitamin B12; 50 mg niacin; 28 mg d-pantothenic acid; 8 mg riboflavin; 0.30 mg selenium; 16 mg Cu from copper sulfate; 110 mg Fe from iron sulfate; 110 mg Zn from zinc sulfate; 33 mg Mn from Manganese sulfate; 0.3 mg I from Ca iodate.

^*d*^Ronozyme HiPhos GT 2700 (DSM Nutritional Products, Basel, Switzerland) provided 2,027 FTU per kg of feed and an expected STTD P release of 0.12%.

^*e*^STTD P = standardized total tract digestible phosphorus.

Based on the results of experiment 1, we were interested in whether the fumonisin products would have better efficacy in diets with lower fumonisin concentrations than those tested in experiment 1. Therefore, we conducted experiment 2 with a lower fumonisin concentration (approximately 30 mg/kg of fumonisin B1 + B2). In experiment 2, a total of 300 pigs (241 × 600; DNA; initially 10.4 kg) were used in a 28-d growth trial with five pigs per pen and 12 replicates per treatment. Experimental treatments were the same as experiment 1, with the exception that the high fumonisin diets only contained approximately 30 mg/kg fumonisin B1 + B2 (Table 2). For experiment 2, a low fumonisin common diet was manufactured identical to the positive control diet in experiment 1 to be used as the positive control (Table 4). The remaining high fumonisin diets (approximately 50 to 60 mg/kg of fumonisin B1 + B2) that were manufactured in experiment 1 were blended with the positive control diet to produce treatment diets with approximately 30 mg/kg of fumonisin (B1 + B2). During blending, each treatment diet was mixed with sand or one of the three commercial products to produce the same product inclusion level as experiment 1. Representative diet samples were obtained from 24 bags per treatment. Pen weights and feed disappearance were measured on d 0, 7, 14, 21, and 28 to determine ADG, ADFI, and G:F. All diets were manufactured at the Kansas State University O.H. Kruse Feed Technology Innovation Center in Manhattan, KS. All diets met or exceeded the NRC (2012) nutrient requirement estimates.

**Table 4.** Diet composition, experiment 2 (as-fed basis)

	Positive control^*a*^	Negative control^*b*^
Item		
Ingredients, %		
Experiment 1: high-fumonisin diet	–	62.50
Positive control^*a*^	–	37.39
Corn	64.70	–
Soybean meal	28.00	–
Soybean oil	3.00	–
Monocalcium phosphate	0.85	–
Calcium carbonate	0.75	–
Sodium chloride	0.60	–
l-Lysine HCl	0.55	–
dl-Methionine	0.21	–
l-Threonine	0.23	–
l-Tryptophan	0.06	–
l-Valine	0.16	–
Vitamin premix^*c*^	0.25	–
Trace mineral premix^*c*^	0.15	–
Phytase^*d*^	0.08	–
Sand	–	0.11
Total	100	100
Calculated analysis		
Standardized ileal digestible amino acids, %		
Lysine	1.30	1.30
Isoleucine:lysine	53	53
Leucine:lysine	111	111
Methionine:lysine	36	36
Methionine and cysteine:lysine	56	56
Threonine:lysine	63	63
Tryptophan:lysine	20.0	20.0
Valine:lysine	69	69
Histidine:lysine	35	35
Net energy, kcal/kg	2,534	2,527
Crude protein, %	19.8	19.8
Calcium, %	0.61	0.61
STTD P^*e*^, %	0.44	0.44

^*a*^The positive control diet was identical to the positive control diet used in experiment 1 and used as blending diet.

^*b*^The three high fumonisin diets with products were manufactured by using the negative control with product added at the expense of sand. 0.11% of Kallsil Dry (Kemin Industries Inc., Des Moines, IA), 0.11% of Feed Aid Wide Spectrum (NutriQuest, Mason City, IA), and 0.06% of Biofix Select Pro (Biomin America Inc., Overland Park, KS).

^*c*^Provided per kg complete feed: 4,133 IU vitamin A; 1653 IU vitamin D3; 44 IU vitamin E; 3 mg vitamin K; 0.03 mg vitamin B12; 50 mg niacin; 28 mg d-pantothenic acid; 8 mg riboflavin; 0.30 mg selenium; 16 mg Cu from copper sulfate; 110 mg Fe from iron sulfate; 110 mg Zn from zinc sulfate; 33 mg Mn from Manganese sulfate; 0.3 mg I from Ca iodate.

^*d*^Ronozyme HiPhos GT 2700 (DSM Nutritional Products, Basel, Switzerland) provided 2,027 FTU per kg of feed and an expected STTD P release of 0.12%.

^*e*^STTD P = standardized total tract digestible phosphorus.

### Serum SA and SO Analysis

Serum samples were collected on d 0, 14, and the last day of trial for experiments 1 and 2 to determine serum SA and SO ratio. For serum samples, two pigs per treatment were bled and serum analyzed as baseline for all treatments on d 0. Nine pigs per treatment were bled and serum analyzed on d 14 and the last day of both trials. For each selected pen, the median weight pig was selected and recorded on d 0. The same pig was used in all serum collection time points. Serum SA and SO was analyzed at the University of Missouri Veterinary Medical Diagnostic Laboratory (Columbia, MO) by HPLC as previously described by Rao et al. (2020). Briefly, whole blood samples were allowed to clot for 30 min, centrifuged at 1,500 × *g* for 15 min and the resulting serum supernatants transferred to polypropylene tubes and stored at −80 °C. Serum samples were sent to the University of Missouri Veterinary Medical Diagnostic Laboratory (Columbia, MO) and analyzed for SA/SO by HPLC with fluorescence detection utilizing a modification of the method of Hsiao et al (2007) and Riley et al. (1994).

### Statistical Analysis

Data were analyzed as a randomized complete block design with block as a random effect and pen as the experimental unit. Models were used to account for heterogeneous variance when appropriate via Bayesian information criterion. Pairwise comparisons were conducted on treatment means using a Tukey adjustment to prevent inflation of type I error due to multiple comparisons. Data were analyzed using nlme package in the R program (R Core Team, 2019). Results were considered significant at *P* ≤ 0.05 and marginally significant at 0.05 < *P* ≤ 0.10.

## RESULTS

### Fumonisin Analysis

For experiment 1, complete diet samples were analyzed twice at NDSU with or without 100× dilution, and once at Trilogy laboratories with 10× dilution. For results from NDSU, the average fumonisin level (B1 + B2) was 61.6 mg/kg when analyzed without dilution, and 56.2 mg/kg when analyzed after dilution. The ratios of fumonisin B1 to B2 before and after dilution were both approximately 4:1 for each individual treatment sample. For the samples analyzed at Trilogy laboratories, the average fumonisin levels were 43.1 mg/kg (B1 + B2) and 47.7 mg/kg (B1 + B2 + B3). The ratio of B1 to B2 was approximately 5.5:1 and consistent for each treatment sample.

For experiment 2, the complete diet samples were analyzed twice at NDSU with 100× dilution, and once at Trilogy laboratories with 10× dilution. The average fumonisin levels (B1 + B2) were 29.3 and 29.5 mg/kg for NDSU, and 23.0 mg/kg for Trilogy laboratories for the high fumonisin diets. The B1 to B2 ratios ranged from 3.2 to 4.1:1 for the first NDSU analysis, 3.4 to 3.8:1 for the second NDSU analysis, and 3.9 to 5.4:1 for the analysis at Trilogy lab.

### Experiment 1

From d 0 to 14 (treatment period), pigs fed the low fumonisin positive control diet or high fumonisin diet with Biofix Select Pro had greater (*P <* 0.05) ADG, ADFI, d 14 BW, and G:F compared to those fed the negative control and high fumonisin diets with Kallsil Dry or Feed Aid Wide Spectrum (Table 5). There was no evidence of differences (*P >* 0.05) between the positive control and high fumonisin diet with Biofix Select Pro for any growth performance criteria. There was no evidence of differences (*P >* 0.05) between the high fumonisin negative control and high fumonisin diet with Kallsil Dry or Feed Aid Wide Spectrum for any growth performance criteria. However, serum SA:SO of all high fumonisin diets were higher (*P <* 0.05) than the positive control with no influence from commercial product inclusion.

**Table 5.** Effect of commercial products on growth performance of nursery pigs, experiment 1^*a*^

			Negative control with			
Item	Positive control	Negative control	Kallsil Dry^*b*^	Feed Aid Wide Spectrum^*c*^	Biofix Select Pro^*d*^	SEM
BW, kg^*e*^						
d 0	9.9	9.9	9.9	9.9	9.8	<0.17^*f*^
d 14	16.7^a^	13.8^b^	13.2^b^	13.6^b^	16.2^a^	0.67
d 27	57.6^a^	50.1^b^	48.0^b^	49.0^b^	56.8^a^	0.95
d 0 to 14 (treatment period)^*g*^						
ADG, g	483^a^	268^b^	228^b^	258^b^	454^a^	14.7
ADFI, g	717^a^	556^b^	506^b^	517^b^	690^a^	17.1
G:F, g/kg	672^a^	480^b^	448^b^	492^b^	659^a^	<25.0^*h*^
d 14 to 27 (post treatment period)^*i*^						
ADG, g	711^ab^	680^bc^	655^c^	666^bc^	736^a^	13.0
ADFI, g	1,184^a^	1,006^b^	984^b^	1,007^b^	1,170^a^	23.3
G:F, g/kg	601^c^	677^a^	668^a^	664^a^	630^b^	7.9
d 0 to 27 (overall period)						
ADG, g	592^a^	461^b^	429^b^	450^b^	590^a^	12.4
ADFI, g	940^a^	767^b^	731^b^	748^b^	921^a^	18.1
G:F, g/kg	629^ab^	601^bc^	586^c^	601^bc^	614^a^	7.2
Mortality, %	0.00	4.44	8.89	6.67	0.00	<4.24^*j*^
Serum SA:SO						
d 14	0.26^b^	1.77^a^	2.15^a^	2.31^a^	1.62^a^	<0.241^*k*^
d 27	0.67	0.61	0.85	0.58	0.50	0.05

^a,b,c^ Means within a row with different superscripts differ (*P* ≤ 0.05).

^*a*^A total of 350 pigs (241 × 600, DNA, Columbus, NE; initially 9.9 kg) were used in a 27-d experiment with 5 pigs per pen and 14 pens per treatment. Means represent 14 pens of pigs per treatment. Means of serum SA:SO represent nine pigs per treatment. Treatment fumonisin (B1 + B2, mg/kg): positive control (4.0), negative control (58.2), negative + Kallsil Dry (54.1), negative + Feed Aid Wide Spectrum (60.8) and negative + Biofix Select Pro (51.8).

^*b*^ Kallsil Dry (Kemin Industries Inc., Des Moines, IA).

^*c*^ Feed Aid Wide Spectrum (NutriQuest, Mason City, IA).

^*d*^ Biofix Select Pro (Biomin America Inc., Overland Park, KS).

^*e*^BW = body weight. ADG = average daily gain. ADFI= average daily feed intake. G:F = feed efficiency. Sa = sphinganine. So = sphingosine.

^*f*^Except pigs in positive control group, all other pigs were fed high fumonisin diets for 14 d.

^*g*^Heterogenous SEM: positive control (0.15), negative control (0.15), Kallsil Dry (0.15), Feed Aid Wide Spectrum (0.15), and Biofix Select Pro (0.17).

^*h*^ Heterogenous SEM: Positive control (6.3), negative control (14.8), Kallsil Dry (14.6), Feed Aid Wide Spectrum (25.0), and Biofix Select Pro (10.9).

^*i*^All pigs were fed low fumonisin common diet for 13 d.

^*j*^ Heterogenous SEM: positive control (0), negative control (3.07), Kallsil Dry (4.24), Feed Aid Wide Spectrum (3.72), and Biofix Select Pro (0).

^*k*^Heterogenous SEM: positive control (0.04), negative control (0.13), Kallsil Dry (0.22), Feed Aid Wide Spectrum (0.24), and Biofix Select Pro (0.20).

During the 13-d posttreatment period, pigs previously fed the high fumonisin negative control diet, or high fumonisin diets with Kallsil Dry or Feed Aid Wide Spectrum had increased (*P <* 0.05) G:F compared with pigs previously fed the low fumonisin diet or high fumonisin diet with Biofix Select Pro. On d 27, the serum SA:SO ratios of pigs fed high fumonisin diets fell below 1.0 and there was no evidence of difference between treatments. Final BW on d 27 BW were approximately 3 kg lighter (*P <* 0.05) when fed the high fumonisin negative control diet or high fumonisin diets with Kallsil Dry or Feed Aid Wide Spectrum compared to pigs fed the positive control diet and high fumonisin diet with Biofix Select Pro.

From d 0 to 27 (overall period), pigs fed the low fumonisin positive control diet or high fumonisin diet with Biofix Select Pro had greater (*P <* 0.05) ADG, ADFI, and d 27 BW compared to those fed the negative control and high fumonisin diet with Kallsil Dry or Feed Aid Wide Spectrum. Pigs fed Biofix Select Pro had improved (*P <* 0.05) G:F compared to those fed the high fumonisin negative control diet or high fumonisin diet with Kallsil Dry or Feed Aid Wide Spectrum. There was no evidence of differences (*P >* 0.05) between the positive control and high fumonisin diet with Biofix Select Pro for any growth performance criteria. There was no evidence of differences (*P >* 0.05) between the high fumonisin negative control and high fumonisin diet with Kallsil Dry or Feed Aid Wide Spectrum on any growth performance criteria.

### Experiment 2

Similar to experiment 1, from d 0 to 28, pigs fed the low fumonisin positive control diet or high fumonisin diet with Biofix Select Pro had greater (*P <* 0.05) ADG, d 28 BW, and G:F compared to those fed the negative control and high fumonisin diet with Kallsil Dry or Feed Aid Wide Spectrum (Table 6). Average daily feed intake was highest (*P <* 0.05) for those fed the diet with Biofix Select Pro compared to the negative control or negative control with Feed Aid Wide Spectrum, with others intermediate. Serum SA:SO measured on d 14 and 28 was lower (*P <* 0.05) for the positive control and pigs fed the diet containing Biofix Select Pro compared to those fed the negative control and high fumonisin diets with Kallsil Dry or Feed Aid Wide Spectrum. There was no evidence of differences (*P >* 0.05) between pigs fed the positive control and those fed the high fumonisin diet with Biofix Select Pro for any growth performance and serum criteria. There was no evidence of differences (*P >* 0.05) between pigs fed the high fumonisin negative control and those fed high fumonisin diet with Kallsil Dry or Feed Aid Wide Spectrum in any growth performance and serum criteria.

**Table 6.** Effect of commercial products on growth performance of nursery pigs, experiment 2^*a*^

			Negative control with			
Item	Positive control	Negative control	Kallsil Dry^*b*^	Feed Aid Wide Spectrum^*c*^	Biofix Select Pro^*d*^	SEM
BW, kg^*e*^						
d 0	10.4	10.5	10.4	10.5	10.4	0.18
d 28	28.4^a^	25.0^b^	25.9^b^	25.4^b^	28.3^a^	0.52
ADG, g	624^a^	518^b^	546^b^	526^b^	626^a^	15.6
ADFI, g	909^ab^	844^b^	866^ab^	845^b^	934^a^	23.2
G:F, g/kg	687^a^	613^b^	631^b^	623^b^	671^a^	6.8
Mortality, %	8.15	0.00	4.01	1.99	1.99	< 4.64^*f*^
Serum SA:SO						
d 14	0.46^b^	1.68^a^	2.02^a^	1.36^a^	0.53^b^	<0.29^*g*^
d 28	0.50^b^	1.51^a^	1.46^a^	1.48^a^	0.54^b^	<0.14^*h*^

^a,b^Means within a row with different superscripts differ (*P* ≤ 0.05).

^*a*^A total of 300 pigs (241 × 600, DNA, Columbus, NE; initially 10.4 kg) were used in a 28-d experiment with five pigs per pen and 12 pens per treatment. Means of growth performance represent 12 pens of pigs per treatment. Means of serum SA:SO represent nine pigs per treatment. Treatment fumonisin (B1 + B2, mg/kg): positive control (4.7), negative control (30.7), negative + Kallsil Dry (31.6), negative + Feed Aid Wide Spectrum (26.7) and negative + Biofix Select Pro (28.5).

^*b*^Kallsil Dry (Kemin Industries Inc., Des Moines, IA).

^*c*^Feed Aid Wide Spectrum (NutriQuest, Mason City, IA).

^*d*^Biofix Select Pro (Biomin America Inc., Overland Park, KS).

^*e*^BW = body weight. ADG = average daily gain. ADFI = average daily feed intake. G:F = feed efficiency. Sa = sphinganine. So = sphingosine.

^*f*^Heterogenous SEM: positive control (4.64), negative control (0), Kallsil Dry (3.12), Feed Aid Wide Spectrum (2.11), and Biofix Select Pro (2.11).

^*g*^Heterogenous SEM: positive control (0.06), negative control (0.16), Kallsil Dry (0.29), Feed Aid Wide Spectrum (0.16), and Biofix Select Pro (0.08).

^*h*^Heterogenous SEM: positive control (0.06), negative control (0.14), Kallsil Dry (0.05), Feed Aid Wide Spectrum (0.12), and Biofix Select Pro (0.06).

## DISCUSSION

Mycotoxins generally have uneven distribution and low concentration in feed ingredients (Zhang et al., 2018), therefore there can be variation during feed manufacturing on dietary fumonisin analysis. Our treatment diets for both experiments were analyzed multiple times for fumonisin levels at North Dakota State University (B1, B2) and Trilogy laboratories (B1, B2, and B3). Although variation in analysis exists between laboratories, the results show the experimental design was successful in creating the desired low and high fumonisin diets with similar fumonisin levels within the high fumonisin diets containing the different products tested. However, the results of the testing indicate the analytical variation that occurs with ingredient sampling and sample preparation.

Increasing prevalence and level of fumonisin contamination in corn has been an emerging issue for swine feed production (Hendel et al., 2020). Although fumonisin has a low absorption rate, can be partially metabolized by porcine microbiota (Fodor et al., 2008; Schertz et al., 2018), and most of the toxin is excreted in feces (Dilkin et al., 2010), high fumonisin concentrations can still cause hepatic and intestinal damage (Motelin et al., 1994; Bouhet and Oswald, 2007; Yuan et al., 2019), and reduce the growth performance of animals (Harvey et al., 2002; Gbore, 2009; Lessard et al., 2009; Rao et al., 2020). The U.S. Food and Drug Administration (FDA) suggests a guidance of maximum fumonisin (B1 + B2 + B3) level of 20 mg/kg in corn used in animal feed. Furthermore, at this level of contamination, the corn should not exceed 50% in swine diets (U.S. Food & Drug Administration, 2001). The European Commission also established a fumonisin recommendation for corn used in animal feed to be below 60 mg/kg of fumonisin (B1 + B2) and no more than 5 mg/kg in complete swine diets (European Commission, 2016). In 2009, EU approved a new functional feed additive group for mycotoxin detoxifying agents (European Commission, 2009); however, these feed additives, which aim to reduce mycotoxin’s negative effects, are not approved to be used as such in the United States. These mycotoxin-detoxifying agents have two main modes of action. They act by either binding the mycotoxins (adsorbing agents) or transforming the mycotoxins into less toxic forms (bio-transforming agents). For the U.S. market, clay based adsorbing agents are the main products used in the animal feed industry and were used in our two experiments.

Adsorbing agents have high molecular weight with specific physical properties (total charge, polarity, and porosity) that bind mycotoxins with hydrophobic binding, hydrogen bonds, electrostatic attraction or repulsion, or coordination bond to form mycotoxin-adsorbing agent complexes. Mycotoxin-adsorbing agent complexes needs to be stable throughout the digestive tract, which has varying pH conditions, in order to reduce the absorption of mycotoxins and eventually be excreted into feces. Different mycotoxins have different physiochemical characteristics (polarity, solubility, and structure). Therefore, one adsorbing agent does not necessarily work with multiple mycotoxins, but usually on one specific kind of mycotoxin. Also, it should be noted that these adsorbing agents, especially activated carbon, may bind to micronutrients, such as minerals and vitamins, therefore reducing the bioavailability of these nutrients (Kolosova and Stroka, 2011). There are several kinds of inorganic and organic adsorbing agents, such as hydrated sodium calcium aluminosilicates (HSCAS), bentonites, zeolites, yeast cell wall, lactic acid bacteria, micronized fiber, activated carbon, organoaluminosilicates, modified clay, and polymers (Vila-Donat et al., 2018). The three commercial products used in these two trials were all categorized as nonmodified clay-based mycotoxin adsorbing agents, which adsorb fumonisin with their physical property and structure (Vila-Donat et al., 2018). Kallsil Dry (Kemin Industries Inc., Des Moines, IA) is labeled as HSCAS and mineral oil. Feed Aid Wide Spectrum (NutriQuest, Mason City, IA) is labeled as bentonite, sodium metabisulfite, and mineral oil. Biofix Select Pro (Biomin America Inc., Overland Park, KS) is a combination of active dry yeast, yeast culture, bentonite, diatomaceous earth, dry kelp, and a natural flavoring compound. Hydrated sodium calcium aluminosilicates can effectively bind with aflatoxins (Kabak et al., 2006; Harper et al., 2010), but failed to be effective on fumonisin or trichothecenes (Vila-Donat et al., 2018). Bentonites are effective for binding aflatoxins and zearalenone (Miazzo et al., 2005; Wang et al., 2012), but in an earlier study was not effective against fumonisin (Avantaggiato et al., 2005). Sodium metabisulfite can restore the performance of pigs from DON contaminated feed (Frobose et al., 2017; Shawk et al., 2018), but no published research has been conducted to test its efficacy against fumonisin. Yeast cell wall has a large capability to adsorb a wide spectrum of mycotoxins, such as aflatoxin, zearalenone, and ochratoxin A (Fruhauf et al., 2012; Pfohl-Leszkowicz et al., 2015), but there is limited information on fumonisin (Pfliegler et al., 2015). Kim et al. (2019) observed that adding a yeast cell wall extract improved ADG for pigs fed diets naturally contaminated with aflatoxin, fumonisin, and deoxynivalenol. Diatomaceous earth restores growth performance and reduces liver damage of broiler chicks fed aflatoxin (Modirsanei et al., 2008; Lakkawar et al., 2017), but no published results can confirm its effect on pigs fed fumonisin contaminated grain. Most studies with fumonisin detoxifying agents have been conducted in vitro, and little in vivo data is available in any animal models, let alone pigs.

The negative effect of fumonisin toxicosis is mainly caused by the competitive inhibition between fumonisin and SA. They have similar molecule structures, which compete for ceramide synthase, an important enzyme for de novo sphingolipid biosynthesis. Therefore, fumonisin disrupts the metabolism of complex sphingolipids that are used in lipid-rich components, such as cell membrane. Without ceramide synthase, the conversion of SA and SO to sphingolipids is disrupted, so SA and SO concentration increases in animal tissue, especially in liver and kidneys. Sphinganine increases at a faster rate than SO, therefore SA:SO ratio has been used as a biomarker for fumonisin toxicosis. Increased SA and SO concentrations are cytotoxic and reduced synthesis of sphingolipids cause cell apoptosis (Merrill et al., 2001). Generally, serum SA:SO ratios above 1:1 are considered fumonisin toxicosis (Hsiao et al., 2007). In experiment 1, even though there was no evidence of difference in growth performance between pigs fed the positive control treatment and those fed high fumonisin diets containing Biofix Select Pro, the high fumonisin still elicited negative effects on d 14 serum SA:SO. This could indicate that SA:SO is a more sensitive indicator of fumonisin contamination than growth performance.

In a previous study, we observed a linear reduction in growth performance when pigs were fed diets contaminated with 7.2 to 35.1 mg/kg of fumonisin (B1 + B2; Rao et al., 2020). Furthermore, serum SA:SO ratios were increased over 1:1 in pigs fed dietary fumonisin level above 32.7 mg/kg. The corn used in the two studies herein came from the same source as the corn used in our previous study, which contained high naturally contaminated fumonisin without other mycotoxins (Tables 1 and 2). This allowed us to determine the effect of fumonisin without the interactive effect of multiple mycotoxins. According to the limited literature on mycotoxin-detoxifying agents on fumonisin, the components of these products do not have well established evidence on binding fumonisin. However, our result suggested that adding Biofix Select Pro in high fumonisin diets reversed the negative effect of fumonisin and had no evidence of difference compared to pigs fed the low fumonisin positive control. The improved performance may due the inclusion of yeast product that may have different properties compared to those in previous research, but the mechanism is unclear. A fumonisin esterase enzyme, which is produced by yeast, has been developed that biotransforms fumonisin into a nontoxic metabolite (European Commission, 2014; Schertz et al., 2018). This enzyme can only be added to diets in purified form in countries where it has been approved. Further research is needed to determine the mode of action of Biofix Select Pro on mitigating the negative effect of fumonisin.

**Figure 1.**
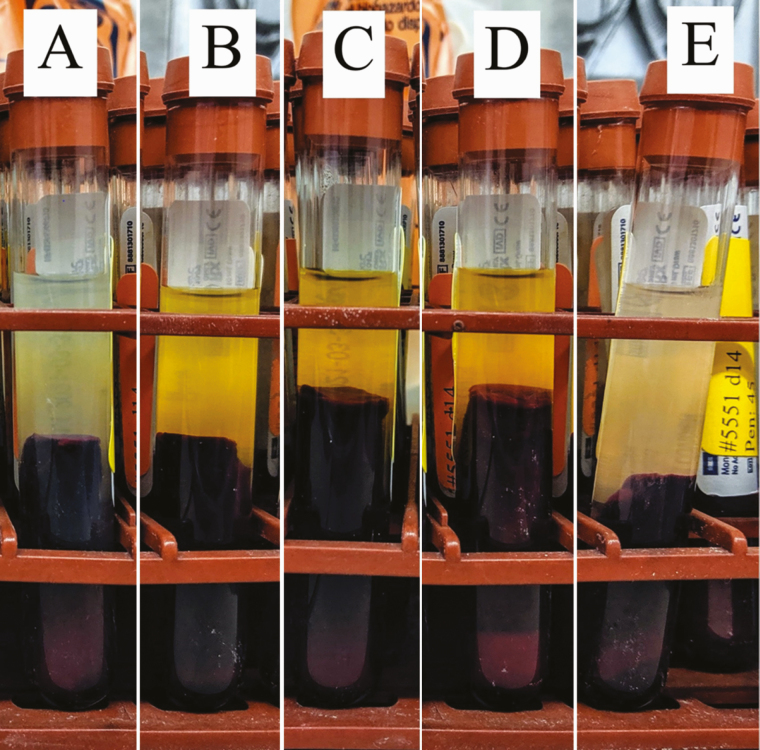
Icteric serum on d 14 of experiment 1. (A) Low fumonisin positive control, (B) high fumonisin negative control, (C) high fumonisin diet with Kallsil Dry, (D) high fumonisin diet with Feed Aid Wide Spectrum, and (E) high fumonisin diet with Biofix Select Pro.

The magnitude of growth reduction caused by fumonisin was different between experiments 1 and 2. Pigs fed 60 mg/kg of fumonisin for 2 wk in experiment 1 had approximately 48% reduction in ADG, while pigs fed 30 mg/kg of fumonisin for 4 wk in experiment 2 had approximately 15% reduction in ADG. However, in a previous study, pigs fed similar concentration of fumonisin as experiment 2 (30 to 35 mg/kg of fumonisin) for 4 wk had approximately 6% reduction in ADG (Rao et al., 2020). We observed icteric serum (Figure 1), a sign of hepatic damage, in pigs fed the high fumonisin negative control diet and high fumonisin diet with Kallsil Dry or Feed Aid Wide Spectrum in experiment 1 (60 mg/kg of fumonisin). Colvin et al. (1993) also observed icterus in pigs fed 200 mg/kg of fumonisin B1 for 4 d and pigs intubated with 4 to 16 mg/kg BW of fumonisin B1. Pigs fed 75 to 100 mg/kg of fumonisin for 1 to 3 wk developed icterus without development of pulmonary edema (Osweiler et al., 1993). Serum of pigs fed the low fumonisin positive control were normal, while serum of pigs fed the high fumonisin diet with Biofix Select Pro were slightly yellow.

### Recovery of Fumonisin Intoxication

In experiment 1, after 14 d of the high fumonisin treatment diets, all pigs were fed a low fumonisin diet in order to observe the recovery from fumonisin contamination. We observed a recovery of growth performance on pigs previously affected by fumonisin, but they did not compensate to the same performance as pigs fed the low fumonisin diet on d 27. The recovery was not just observed on growth performance, but also in SA:SO ratios. Icteric serum, which was observed in d 14 serum samples, was not observed in d 27 serum samples. The d 27 serum SA:SO ratios were reduced to below 1:1 ratio indicating that there was minimal fumonisin intoxication. These results suggest that pigs experienced a recovery from fumonisin toxicosis after a short-term (14 d) exposure to 50 to 60 mg/kg of fumonisin; however, the pigs did not recover to similar BW as the positive control in the 13-d posttreatment period. Whether pigs previously affected by high fumonisin can recover to the same performance as non-affected pigs needs further research. These results match the literature on fumonisin metabolism. The biodistribution and pharmacokinetics of fumonisin in pigs indicates a rapid absorption and rapid elimination through feces, but a low residue would remain in tissue, especially in liver and kidneys because of their high affinity toward fumonisin. This long half-life is primarily attributed to the enterohepatic recirculation of fumonisin (Prelusky et al., 1994, 1996). Therefore, withdrawing fumonisin contaminated feed from pigs previously fed 14 d of high fumonisin (60 mg/kg) and offering a diet low in fumonisin would reduce the negative effect of fumonisin within a short period of time (13 d), but pigs may still have a low tissue residual that does not have evidence to affect pig’s serum SA:SO ratio. Also, the length of recovery period for pigs fed high fumonisin for more than 14 d needs further research.

In conclusion, feeding high fumonisin contaminated diets reduced growth performance and increased the SA:SO ratio. Adding Biofix Select Pro to diets appeared to mitigate the negative effects of high fumonisin concentrations, while Kallsil Dry and Feed Aid Wide Spectrum did not. The SA:SO ratio appears to be a sensitive indicator of fumonisin contamination.
